# Mechanisms of drought tolerance in legumes: physiological impacts, adaptive responses, and phenotyping strategies

**DOI:** 10.3389/fpls.2026.1844285

**Published:** 2026-07-21

**Authors:** Andrea Fernandez-Gutierrez, Alvaro F. Rodriguez-Torres, Antonio Encina, Juan J. Gutierrez-Gonzalez

**Affiliations:** 1 Área de Genética, Departamento de Biología Molecular, Universidad de León, León, Spain; 2 Área de Fisiología Vegetal, Departamento de Ingeniería y Ciencias Agrarias, Universidad de León, León, Spain; 3 Instituto de Biología Molecular, Genómica y Proteómica (INBIOMIC), Universidad de León, León, Spain

**Keywords:** drought signaling, drought-tolerance assessment, high-throughput phenotyping, legume abiotic stress, water stress physiology, legume drought stress

## Abstract

Drought is recognized as the primary abiotic stress limiting global crop productivity and poses a significant threat to food security. Consequently, the genetic improvement of drought tolerance has become a priority for modern plant breeding. Developing resilient cultivars requires a fundamental understanding of the physiological, biochemical, and molecular mechanisms that plants employ to counteract water deficits. This review provides a comprehensive analysis of drought-induced effects across various developmental stages in legumes, detailing the signaling networks that facilitate stress perception and response. Furthermore, we evaluate the experimental parameters and methodologies frequently used to assess drought tolerance, weighing their respective advantages and limitations. Finally, we analyze the revolutionary role that high-throughput phenotyping could play in stress assessment and precision breeding.

## 1 Introduction

Legumes, a diverse group of plants belonging to the family Fabaceae, are essential components of human and animal nutrition, serving as a primary source of plant-based protein and fiber. Chickpeas, lentils, beans, peas and cowpeas are central to global diets, especially vegetarian and vegan diets, where they serve as a primary source of plant-based protein (Semba et al., 2021). While other legumes, such alfalfa or soybeans, are vital for livestock feed. Beyond their nutritional value, legumes enhance soil fertility through symbiotic N_2_ fixation, which reduces reliance on synthetic fertilizers and promotes sustainable agricultural rotations (Ferreira et al., 2021; Jimenez-Lopez et al., 2023). Driven by the rising demand for non-animal protein sources, the legume market is projected to expand significantly in the coming years.

In fact, legumes are positioned as one of the best alternatives, as they are an economical, versatile, and environmentally responsible source of protein (Ferreira et al., 2021; Stagnari et al., 2017). However, legume production faces a significant threat from drought - a water deficit resulting from abnormally low precipitation that severely compromises plant fitness. Climate change is expected to increase both the frequency and intensity of drought events (IPCC, 2023), further endangering agricultural stability. Drought disrupts key physiological processes, leading to diminished seed quality and substantial yield losses, particularly during critical reproductive stages (Gutierrez-Gonzalez et al., 2010; Pérez de la Vega et al., 2022; Morgil et al., 2017; Yang et al., 2021). Not surprisingly, drought is considered the most harmful stress for legume crops (Pérez de la Vega et al., 2022; Khatun et al., 2021). Tolerance involves signaling cascades that trigger morphological, physiological, and biochemical adaptations via the up- or down-regulation of specific genes (Fernandez-Gutierrez et al., 2026a; Hussain et al., 2019).

While drought tolerance is a complex trait with a strong genetic component (Fernandez-Gutierrez et al., 2026b; Jha et al., 2020), the mechanisms by which tolerant accessions withstand these conditions remain largely unidentified, although several QTLs and linked markers have been identified in different legume species (**Table 1**). Wild relatives often harbor beneficial alleles for stress resilience that are absent in modern cultivars (Gutierrez-Gonzalez et al., 2022), offering a vital resource for breeding programs.

**Table 1:** QTLs and markers found associated with drought tolerance in legumes.

Specie	QTL/marker associated with drought tolerance	References
*A. hypogaea*	qHW-A01.1, qHW-A05.4, qHW-B01.4, qHW-B09.1, qPW-A03.1, qPW-A02.1, qPW-A03.2, qSW-A03.1, qDW-A05.3, qDW-A05.2, qSLA-A03.4, qSLA-A04.5, qISC-A04.1, qTR-A09.1, qNB-A07.1, qSCMRd-A04.4, qHW-A03.1, qHW-A03.2, qTE-A03.1, qTE-A03.2, qDW-A05.1, qHW-A05.1, qTE-A07.1, qDW-B01.1, qDW-B01.2, qPW-B01.1, qPW-B01.2, qSW-B01.1, qSW-B01.2, qTR-B01.1, qHW-B09.1, qHW-B09.2	(Pandey et al., 2020)
*C. arietinum*	RLD4, RSA6, RDWR4	(Jaganathan et al., 2014)
*C. arietinum*	qYLD7.1, qRWC1.1	(Yadava et al., 2023)
*C. arietinum*	LG3, LG4	(Hamwieh et al., 2013)
*C. arietinum*	TAA170, ICCM0249, GA24, STMS11, NCPGR21, NCPGR127, GA11, TR11	(Bharadwaj et al., 2021)
*G. max*	qWDC1-1, qWDC1-2, qWDC6-1, qWDC7-1, qWDC7-2, qWDC7-3, qWDC10-1, qWDC10-2, qWDC19-1	(Park et al., 2025)
*G. max*	qPN-WW19.1, qPN-DS8.8, qBM-WW1, qBM-DS17.4, qSW-WW4 and qSW-DS8	(Li et al., 2023)
*G. max*	qWS-11-2, qDTW-11-2	(Jiang et al., 2024)
*G. max*	FR_Gm01, FR_Gm03, FR_Gm04	(Abdel-Haleem et al., 2010)
*L. culinaris*	nsd-1, yld-1, nsd%-1, rwc%-1, rwc-1, nsd-2, rwc%-2, hsw-1, hsw%-1, nsd%-2, yld%-1 , hsw-2, nsd-3, yld-2, hsw%-2, hsw%-3, yld%-2, yld-3, hsw-3, nsd-4, hsw%-4, hsw-4, hsw%-5, hsw-5, yld-4, nsd-5	(Fernandez-Gutierrez et al., 2026b)
*L. culinaris*	Chr2:206859313, Chr4:476664664	(Kumar et al., 2025)
*P. sativum*	Pv03	(Mukeshimana et al., 2014)
*P. sativum*	A6, AA175, AC74, AD57, AB141, AB64, Psblox2, PsAAP2_SNP4, DipeptIV_SNP1	(Iglesias-García et al., 2015)
*P. sativum*	M-QTL1.1, M-QTL2.1, M-QTL3.1, M-QTL4.2, M-QTL5.1, M-QTL7.1, M-QTL7.3	(Jha et al., 2025)
*P. vulgaris*	SY1.1PR, SY2.2BR, SY3.3PR, SY3.4PR, SY4.1PR, SY6.1PR	(Dramadri et al., 2019)
*V. faba*	qCT-2012, qCT-2013, qCT-drought-2013, qSD-2013-1, SL-2013, qgs2013, qSD-2014, qCT-2014	(Khazaei et al., 2014)
*V. unguiculata*	Dro-1, Dro-2	(Muchero et al., 2009)

Breeding improved drought-tolerant legumes is essential for sustaining global food supplies under future climate scenarios. Plant breeding is most effective when the mechanisms underlying the desired traits or phenotypes are well understood (Varshney et al., 2021). Consequently, developing drought-tolerant legume varieties requires a four-pronged approach: first, characterizing the impacts of drought stress on plant development and crop yield; second, understanding how tolerant accessions mitigate these effects; third, identifying the genetic factors (*loci*) and molecular markers that facilitate the tolerance response; and finally, introgressing these genetic factors into elite cultivars.

Research on drought tolerance in legumes remains limited, with soybeans currently being the most extensively studied species. While the primary molecular pathways involved in activating drought tolerance responses in legumes have been identified, a comprehensive review of these pathways is still lacking. This review aims to provide an exhaustive analysis of the morphological, physiological, and biochemical effects triggered by water stress in legume crops, along with their corresponding response mechanisms. We also summarize the morphological, physiological, and biochemical parameters used to assess drought tolerance in legumes, including recent advances in high-throughput phenotyping (HTP).

## 2 Effects of drought stress on legumes

**Figure 1:**
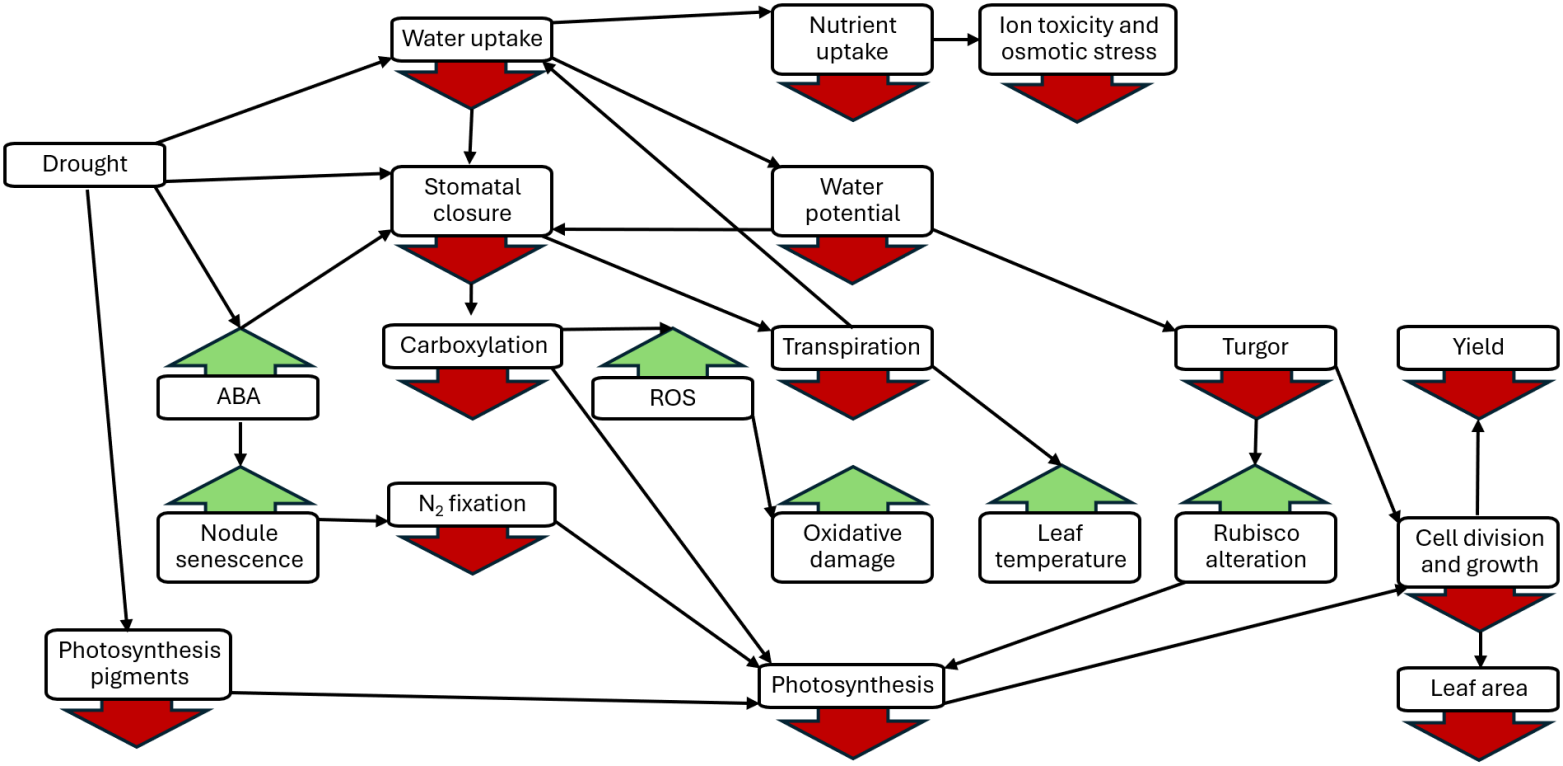
Major effects of drought stress in legumes. The interactions and feedback between the different physiological responses that occur under water deficit conditions are illustrated. Processes or responses that are induced/enhanced (green arrows) or reduced/inhibited/suppressed (red arrows) are indicated. Authors’ own elaboration.

Low water availability significantly alters key morphological, physiological, and biochemical processes (summarized in **Figure 1**). Plant responses span from germination through flowering and typically involve a “domino effect” of events that, in legumes, results in reduced yields and poor seed quality (Seleiman et al., 2021).

Drought stress initially arrests or delays seed germination and can produce weakened seedlings (Khatun et al., 2021). Water scarcity inhibits enzymatic functions, thereby hampering protein hydrolysis, reserve mobilization, and energy production, which ultimately impairs germination (Muscolo et al., 2014). In chickpeas, early drought stress has been shown to severely reduce germination rates (Yücel et al., 2010). Furthermore, when stress occurs during the seedling stage, it decreases growth rates, particularly in susceptible genotypes of legumes such as peas and soybeans (Jaybhaye et al., 2024; Tamindžić et al., 2024).

Following seedling establishment, an immediate consequence of drought stress is stomatal closure, which reduces transpiration to counteract water loss (**Figure 1**). Stomatal closure may occur in response to low turgor and/or low leaf water potential (Buckley, 2019). However, recent studies suggest this response may be linked more closely to low soil moisture than to leaf water potential (Abdalla et al., 2021; Carminati and Javaux, 2020). Current evidence indicates that stomatal closure is associated with hydraulic signals arising along the soil-plant continuum and may begin before leaf turgor loss becomes severe (Cardoso et al., 2025). Specifically, rhizosphere drying and declining root-soil hydraulic conductivity appear to contribute significantly to the early onset of stomatal closure during soil drying (Di Bert et al., 2026).

By limiting water loss, stomatal closure also triggers a rise in leaf temperature (Fahad et al., 2017). Furthermore, it entails a reduction in stomatal conductance (Pang et al., 2017a), which lowers CO_2_ influx and, consequently, carboxylation (Asargew et al., 2024; Gahir et al., 2021).

Reduced transpiration also leads to diminished water uptake from the soil and a decrease in internal plant water content (Wahab et al., 2022). Soil water intake is coupled with nutrient uptake, which is also highly sensitive to soil moisture levels. Soil drying reduces root-soil hydraulic conductivity and nutrient mobility, further restricting the plant’s ability to acquire water and nutrients (Di Bert et al., 2025; Holz et al., 2024). Nutrient diffusion to the roots becomes impossible below certain moisture thresholds, and transport is severely hampered by low water potentials (Khatun et al., 2021). Additionally, the loss of soil moisture can lead to the accumulation of salts and ions in the upper soil layers - where the majority of roots are located - resulting in osmotic stress and ion toxicity (Ma et al., 2020). This decline in leaf water and osmotic potential is commonly associated with reduced turgor pressure and relative water content, thereby disrupting normal plant-water relations (Berauer et al., 2024). In lentils, osmotic adjustment has been identified as a key physiological response to drought, with significant genotypic variation observed among accessions under water deficit (Ashraf et al., 1992; Noor et al., 2024).

Drought impacts all components of the photosynthetic process, leading to limited production of photosynthates and energy (Qiao et al., 2024). Beyond stomatal limitation, sustained drought imposes non-stomatal constraints on photosynthesis, including photoinhibition, damage to photosystems, and restrictions in electron transport and C assimilation (Qiao et al., 2024). A reduction in plant water content leads to a loss of turgor, hampering cell division and growth (Thapa et al., 2011). This decrease in turgor also causes a reduction in chloroplast volume, which alters the conformation of Rubisco and impairs its functionality (Thapa et al., 2011). Furthermore, reduced growth restricts leaf area, subsequently lowering chlorophyll content and limiting light harvesting (Khatun et al., 2021). Non-cyclic electron transport and ATP synthesis are similarly affected. Drought exposure also enhances the photo-oxidation of photosynthetic pigments while inhibiting their biosynthesis (Hussain et al., 2019). In parallel, drought typically accelerates chlorophyll degradation and enhances non-photochemical quenching through the xanthophyll cycle as a photoprotective response; however, prolonged drought stress eventually lowers photochemical efficiency (Wang et al., 2025). Consequently, limited photosynthesis leads to an increased concentration of reactive oxygen species (ROS), ultimately resulting in oxidative stress.

ROS are toxic, highly reactive molecules that damage lipids, carbohydrates, proteins, and DNA. The primary ROS molecules include the hydroxyl radical, hydrogen peroxide, singlet oxygen, and the superoxide anion radical. Under drought conditions, chloroplasts are major intracellular sources of ROS, although peroxisomes and mitochondria also contribute. Importantly, chloroplast-derived ROS function not only as damaging agents but also as signaling molecules that activate stress responses (Lee and Kim, 2024). A critical effect of ROS-mediated oxidative damage is the peroxidation of lipid membranes, which jeopardizes membrane integrity (Khatun et al., 2021). ROS can also influence protein redox status, causing functional changes and modifications in transcription and translation (Ilyas et al., 2021). Furthermore, limited carboxylation results in an accumulation of reduced nicotinamide adenine dinucleotide (NADPH), leading to a scarcity of NADP+. Consequently, free electrons are diverted toward ROS formation (Khatun et al., 2021). Additionally, the increase in internal O_2_ relative to CO_2_ enhances photorespiration. While this aids in antioxidant defense, it also amplifies oxidative stress by elevating hydrogen peroxide production in the peroxisomes (Farooq et al., 2017).

These physiological and biochemical disruptions eventually trigger morphological changes. Under water stress, the subsequent loss of turgor decreases cell elongation, division, and differentiation, leading to significant growth reduction (Thapa et al., 2011). This inhibition occurs primarily in the leaves (Hussain et al., 2019) and, alongside changes such as rolling and wilting, can ultimately lead to leaf abscission if stress is prolonged (Khatun et al., 2021; Nadeem et al., 2019). Furthermore, overall plant height and biomass production are diminished due to mitotic decline and limited dry matter production via photosynthesis. In N_2_-fixing legumes such as soybeans, these effects may be exacerbated by reductions in symbiotic N_2_ fixation and drought-induced shifts in source-sink relationships, which restrict the supply of C and N to developing tissues (Rubia et al., 2025).

Reproductive stages, such as flowering and seed filling, are the most vulnerable phases of legume development to water deficit (Gutierrez-Gonzalez et al., 2010; Khatun et al., 2021; Nadeem et al., 2019). When water stress occurs at pre-anthesis, the time to flowering is often reduced, whereas post-anthesis stress shortens the seed-filling period (Farooq et al., 2017). Under water scarcity, both flower formation and gametogenesis are impaired, leading to pollen sterility (Farooq et al., 2017). This accelerated flowering time, coupled with drought-limited photosynthesis, results in smaller flowers that lack sufficient photosynthates for optimal embryo development (Khatun et al., 2021). Consequently, the number of viable flowers decreases compared to plants grown under well-watered conditions. Furthermore, the number of pods that successfully initiate development is reduced, and many undergo abortion, primarily due to the limited sink capacity of reproductive organs under drought stress (Farooq et al., 2017; Poudel et al., 2025). This reduction in photosynthate availability varies by pod position; for instance, in soybeans, distal flowers exhibit lower pod set under drought compared to proximal pods (Kokubun and Honda, 2000).

The morphological, biochemical, and molecular effects of drought collectively diminish seed production and overall yield (Khatun et al., 2021). The duration and developmental stage at which stress occurs determine the severity of these losses (**Table 2**). For example, drought during the vegetative stage can lead to yield losses of approximately 70% in cowpeas (Nunes et al., 2022). Impact during the podding stage is even more severe, potentially causing over 80% yield loss in chickpeas (Pang et al., 2017b) and nearly 80% losses in lentils (Sehgal et al., 2017). While water stress at the germination and seedling stages affects yield by reducing the final plant population, stress at vegetative stages weakens plant fitness through disrupted photosynthesis and altered dry matter allocation. Nevertheless, the most devastating impacts on yield occur during pod setting and seed filling. At these stages, drought compromises both the quantity and the quality of the seeds produced (Khatun et al., 2021). Specifically, drought stress during pod-filling triggers the accumulation of abscisic acid (ABA) in young chickpea pods, eventually leading to their abscission (Pang et al., 2017b).

**Table 2:** Drought yield losses on different legume crops.

Grain legumes	Phenological stage	Yield lsses (%)	References
Chickpea (*Cicer arietinum* L.)	Flowering	27–40	Mafakheri et al. (2010)
	Reproductive	45–69	Nayyar et al. (2006)
	Podding	79-94	Pang et al. (2017b)
Common bean (*Phaseolus vulgaris* L.)	Flowering	49	Rosales-Serna et al. (2004)
	Reproductive	58–87	Martínez et al. (2007)
	Podding	40	Ghanbari et al. (2013)
Cowpea (*Vigna unguiculata* L.)	Vegetative and reproductive	70	Nunes et al. (2022)
	Reproductive	40-56	Ahmed and Suliman (2010)
	Podding	29	Kyei-Boahen et al. (2017)
Lentil (*Lens culinaris* Medik.)	Reproductive	24	Allahmoradi et al. (2013)
	Grain filling	50-78	Sehgal et al. (2017)
Soybean (*Glycine max* L.)	Podding	45–82	Wei et al. (2018)
	Grain filling	42	Maleki et al. (2013)

Modified from Fernandez-Gutierrez et al., 2026a.

### 2.1 Role of symbiotic relations in modulating drought stress

A principal feature inherent to legumes is their ability to form symbiotic relationships with soil microbes. Legumes establish symbiosis principally with three types of microorganisms: rhizobia (N_2_ -fixing bacteria), nodule non-rhizobial bacteria (NRB), and arbuscular mycorrhizal fungi (AMF), which enhance the plant survival in different conditions, including water scarcity (Álvarez-Aragón et al., 2023; Etesami and Santoyo, 2025; Hu et al., 2022). Rhizobia, NRB, and AMF not only improve legume performance alone but also through synergistic relationships. Rhizobia provide fixed N to the plant, AMF act as a reservoir of this N and enhance phosphorus uptake, while nodule NRB enhance nodule performance. Together, these microorganisms provide nutrients for the plant to grow even under water scarcity situations (Duan et al., 2024; Etesami and Santoyo, 2025).

Under drought stress, nodulating legumes show better performance suggesting a protective role of nodule-associated bacteria (mainly rhizobia). Rhizobium symbiosis allows the maintenance of water status and reduces oxidative damage (Álvarez-Aragón et al., 2023). Furthermore, expression of stress related genes and biochemical stress markers such as proline or dehydrins was found to be weaker in nodulated plants, revealing an improved physiological state (Álvarez-Aragón et al., 2023). In kidney beans, for example, the inoculation of a certain type of rhizobia resulted in an improved tolerance to drought stress, promoting the expression of drought-related genes, as well as enhancing antioxidant activity and growth (Li et al., 2025). Within nodules, rhizobia were found to take part of a complex microbiome in which several non-rhizobial microorganisms establish beneficial relations among them and with the plant (Martínez-Hidalgo and Hirsch, 2017; Etesami and Santoyo, 2025). Under drought conditions, nodule NRB may contribute to maintaining nodule functionality by modulating phytohormone homeostasis, and mineral nutrient availability, which can help sustain effective legume–rhizobium symbiosis (Etesami and Santoyo, 2025).

AMF has proven to be beneficial for legume drought tolerance. The AMF hyphae constitute a vast network able to reach the soil layers where roots were not capable to extend. Through this hyphal network water can be transported to the plant allowing the obtention of water resources under deficits. Furthermore, AMF growth also contributes to soil structure and water capacity retention (Hu et al., 2022).

### 2.2 Nodulation and symbiotic nitrogen fixation under drought stress

Nodulation and symbiotic N_2_ fixation are highly sensitive to water deficit in legumes. Drought can reduce nodule formation, growth, and function by affecting root and nodule water status, O_2_ diffusion, rhizobium metabolism, and nitrogenase activity (Serraj et al., 1999; Arrese-Igor et al., 2011; Gorim and Vanderberg, 2017; Iqbal et al., 2022). Because biological N_2_ fixation is energy-intensive, drought-induced reductions in photosynthesis and C transport can lead to strong C/N trade-offs, forcing the plant to redistribute limited C resources between root and stem growth, drought responses, and nodule metabolism. Consequently, the C and energy available to maintain bacteroid respiration, nitrogenase activity, and ammonium assimilation may decline, reducing symbiotic N_2_ fixation and the plant’s nitrogen nutrition (Liu et al., 2018; Schwember et al., 2019; Ferguson et al., 2019; Nadeem et al., 2019; Khatun et al., 2021; Petrushin et al., 2023; Rubia et al., 2025). The magnitude of this inhibition depends on the legume species, the type of nodule, the stage of development, and the rhizobium strain, as strains differ in nodulation competitiveness, stress tolerance, and N_2_ fixation efficiency under unfavorable soil conditions (Ji et al., 2017; del-Canto et al., 2023).

Prolonged drought also accelerates nodule senescence through several interconnected mechanisms, involving oxidative stress and redox imbalance, defense-related signaling, loss of nodule functionality, and reduced N_2_ fixation capacity (Dhanushkodi et al., 2018; Zhou et al., 2021; Berrabah et al., 2024; da Silva et al., 2024; Rubia et al., 2025). Therefore, delaying or modulating drought-induced nodule senescence has been proposed as a promising strategy for maintaining legume productivity under water stress (Zhou et al., 2021).

Although direct yield responses depend on many factors, maintaining functional nodulation under drought is expected to contribute to yield stability by sustaining N_2_ fixation and N nutrition (Satognon et al., 2026). In this context, plant growth-promoting bacteria, including rhizobia and NRB, can mitigate the effects of drought through various mechanisms, such as improving nutrient bioavailability, stimulating root growth, increasing nodulation, and modulating stress-related hormonal responses (Saikia et al., 2018; Namwongsa et al., 2019; Alemneh et al., 2020). In particular, ACC deaminase-producing bacteria have been shown to reduce water stress-induced ethylene accumulation, facilitating nodulation and improving legume growth under adverse conditions (Gamalero and Glick, 2025). Beyond microbial inoculation, microbiome engineering is emerging as a complementary strategy to optimize plant-microbe interactions by manipulating rhizosphere traits, root exudation, or microbial community composition, with the aim of improving nutrient uptake, stress resilience, nodulation, and symbiotic performance under drought conditions (Backer et al., 2018; Joshi et al., 2025; Chesneau et al., 2025).

## 3 Water stress signaling

Under abiotic stresses such as drought, signaling molecules play a central role in transducing stress perception from the roots to other plant organs and coordinating appropriate adaptive responses (Chhaya et al., 2021). The mechanism of action of phytohormones and other signaling molecules have been studied primarily in non-legume species. However, their conserved functions allow this knowledge to be applied to legumes (Kurepa and Smalle, 2025). Early drought perception involves fluctuations in cellular turgor and hydraulic signals, which rapidly induce cytosolic Ca²^+^ transients and reactive oxygen species (ROS) production. They act as secondary messengers in stress signaling (Takahashi et al., 2020), triggering downstream signaling cascades and transcriptional reprogramming, propagating the signal systemically throughout the plant via hydraulic and ROS/Ca²^+^-mediated pathways (Takahashi et al., 2020).

### 3.1 Phytohormonal signaling of water stress

ABA is regarded as the most critical phytohormone for abiotic stress tolerance, including drought, heat, cold, and salinity (Petrushin et al., 2023). ABA is synthesized in various plant organs, but predominantly in the roots. Its accumulation is further modulated by long-distance root-to-shoot signaling mechanisms that can induce ABA biosynthesis in the leaves, specifically through the NCED enzymatic activity (Zhang et al., 2018; Kuromori et al., 2018). Briefly, increased ABA concentration in the leaves triggers stomatal closure, enhances root-to-shoot ratios by inhibiting leaf expansion and aerial growth, and activates the synthesis of late embryogenesis abundant proteins, antioxidants, dehydrins, and other protective proteins (Ahmad et al., 2023; Aslam et al., 2022). Furthermore, ABA accumulation promotes the synthesis and deposition of cuticular wax, limiting stomata-independent water loss (Xue et al., 2017). At the signaling level, ABA responses are primarily mediated by the PYR/PYL/RCAR-PP2C-SnRK2 pathway, which is central to drought signal transduction (Cutler et al., 2010). At the transcriptional level, drought signaling converges on several key transcription factor families, including bZIP/ABF, DREB/ERF, NAC, WRKY, and MYB. They integrate hormonal and redox signals to regulate stress-responsive gene expression (Nakashima and Yamaguchi-Shinozaki, 2013; Yoshida et al., 2014; Zha et al., 2025).

Other phytohormones have recently gained attention for their roles in drought tolerance. Among them, jasmonates, including jasmonic acid, methyl jasmonate, and jasmonoyl-isoleucine, are synthesized in flowers and regulate processes such as pollen production and fruit ripening (Ahmad et al., 2023). Under drought conditions, jasmonates have been linked to stomatal closure, root development, and ROS detoxification (Ahmad et al., 2023), presumably by acting through the MYC family of transcription factors (Riemann et al., 2015). Furthermore, an increase in jasmonate concentration appears to elevate ABA levels. Jasmonates have also been shown to improve root hydraulic conductivity, thereby promoting water uptake under limited soil moisture (Chhaya et al., 2021).

Ethylene is another phytohormone critical to stress tolerance. Ethylene signaling operates through the AP2/EREBP family of transcription factors (TFs) and can activate DRE/CRT cis-elements to promote drought-mitigating responses (Müller and Munné-Bosch, 2015). Ethylene participates in drought signaling in a doses-dependent manner. Under moderate or transient water deficit, ethylene may contribute to acclimation by modulating root growth, source-sink dynamics, photosynthetic performance, and antioxidant or osmoprotective responses (Müller and Munné-Bosch, 2015; Nazir et al., 2024). However, under severe drought, high ethylene concentrations are often associated with growth inhibition, accelerated senescence, chlorophyll degradation, and leaf or organ abscission (Müller and Munné-Bosch, 2015; Nazir et al., 2024). In legumes, this senescence-related role is also relevant to nodules, where stress and defense signaling intersect with nodule aging and functional decline (Berrabah et al., 2024).

Salicylic acid (SA) is involved in leaf transpiration, flowering, chlorophyll metabolism, antioxidant defense, and osmotic adjustment. Low concentrations of SA are thought to strengthen drought tolerance by promoting enzymatic antioxidant activity, whereas higher concentrations can induce oxidative stress (Ahmad et al., 2023; Kumar Patel et al., 2011; Melo et al., 2024). In chickpeas, foliar application of low concentrations of SA improved grain yield under drought conditions. However, higher SA concentrations resulted in values similar to those observed for the controls (Melo et al., 2024). This protective effect appears to be associated with an increase in proline and other osmolyte concentrations, contributing to maintaining cell turgor (Chhaya et al., 2021). In addition, the accumulation of SA has been shown to increase ABA sensitivity, promoting stomatal closure thereby amplifying its physiological effects (Salvi et al., 2021).

Auxins also contribute to the activation of drought responses, even though their synthesis typically decreases under water deficit as ABA and ethylene levels rise. For instance, under drought conditions, auxins can stimulate ROS production, which serves to “prime” the plant’s antioxidant defenses. They also upregulate other stress-responsive hormones, such as ABA and jasmonates (Chhaya et al., 2021). Additionally, auxins are key regulators of root system architecture. Rather than acting solely through ROS induction, auxin responses are largely mediated by changes in spatial distribution and transport, which regulate lateral root formation and root elongation, ultimately improving water foraging capacity (Dinneny, 2019).

Cytokinins are implicated in the regulation of key biological processes, including adaptation to low water conditions. They enhance drought tolerance through both up- and down-regulation mechanisms. Cytokinins are responsible for maintaining apical dominance in roots and promoting primary root growth. Their balance with auxin concentrations dictates the promotion or inhibition of lateral root formation, making both phytohormones essential for determining root system architecture (Chhaya et al., 2021). Furthermore, high cytokinin concentrations in leaves reduce sensitivity to ABA, leading to stomatal opening. This can improve photosynthesis and mitigate oxidative damage under certain conditions (Hai et al., 2020). The capacity of cytokinins to regulate stomatal conductance suggests an essential role in modulating water balance (Ahmad et al., 2023). While the precise underlying mechanisms remain partially elusive, the role of cytokinins in drought tolerance appears to be mediated by extensive crosstalk with other phytohormones (Ahmad et al., 2023).

In contrast to auxins and cytokinins, gibberellins (GAs) generally act as negative regulators of drought tolerance. A drought-induced reduction in GA levels promotes the accumulation of proline and other amino acids involved in ion transport, detoxification, and osmoregulation (Liao et al., 2025). GAs regulate developmental processes primarily through the repression of DELLA proteins. Under drought stress, the reduction in GA content allows for the stabilization of DELLAs, which then interact with several transcription factors to restrain vegetative growth. Furthermore, DELLA proteins have been shown to feedback-regulate GA content, modulating the balance between defense and growth responses (Sarwar et al., 2023). Low GA concentrations have been associated with suppressed plant growth, smaller leaves, and higher stomatal densities coupled with low stomatal conductance, all of which are recognized as drought-tolerant traits (Ahmad et al., 2023; Chhaya et al., 2021).

### 3.2 Phytohormone crosstalk in drought stress responses in legumes

**Figure 2:**
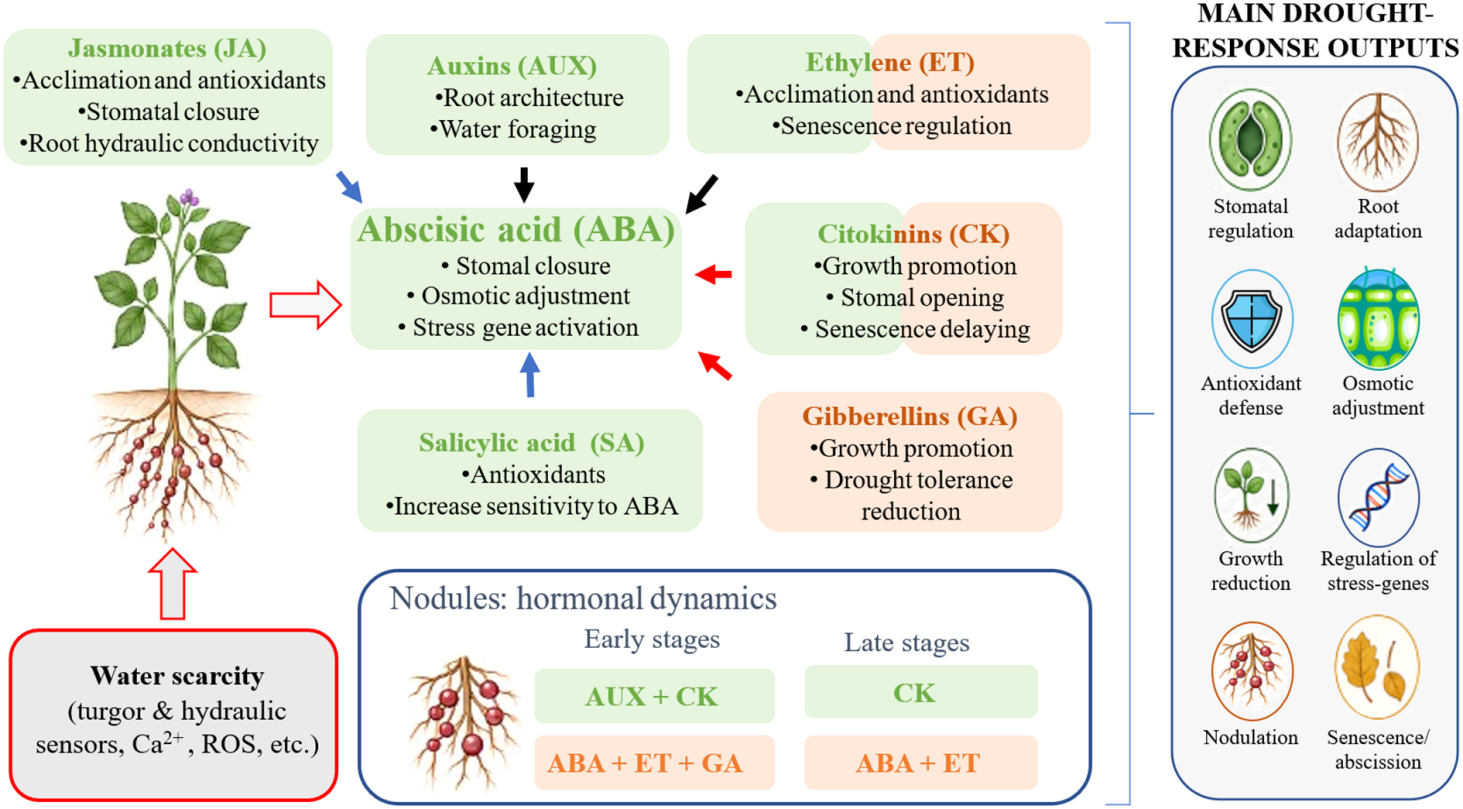
Overview of phytohormone interactions during drought stress in legumes. Water stress signaling is regulated by a dynamic network of phytohormones involving abscisic acid (ABA), jasmonates (JA), auxins (AUX), gibberellins (GA), salicylic acid (SA), cytokinins (CK) and ethylene (ET). The outcome of phytohormone activity is strongly context-dependent (concentration, tissue, developmental stage, and stress intensity) and governed by crosstalk networks. Nevertheless, their main role as positive (green boxes) or negative (orange boxes) regulators of drought tolerance is indicated. The main interactions with ABA are described as synergistic (blue arrows), antagonistic (red arrows), or context-dependent (black arrows). The bottom panel summarizes the phytohormone regulation of nodule initiation and development under drought stress. Positive regulators (green boxes) and negative regulators (orange boxes) have been highlighted. Adapted and summarized from Cutler et al. (2010); Müller and Munné-Bosch (2015); Riemann et al. (2015); Hai et al. (2020); Chhaya et al. (2021); Salvi et al. (2021); Ahmad et al. (2023); Shaffique et al. (2023); Berrabah et al. (2024); da Silva et al. (2024); Nazir et al. (2024), and Etesami and Santoyo (2025). Author’s own elaboration.

Phytohormone signaling under drought should be interpreted as an integrated regulatory network rather than as isolated pathways (**Figure 2**). Recent research on soybean has highlighted that, despite the predominant role of ABA, water stress responses in legumes are governed by a dynamic hormonal network involving ABA, jasmonates, GAs, SA, cytokinins, auxins, and ethylene (Shaffique et al., 2023). Overall, these phytohormones constitute a complex regulatory network that senses water-deficit signals within the plant’s environment acting cooperatively to regulate drought stress responses (Sirhindi et al., 2021).

Jasmonates can act synergistically with ABA by contributing to stomatal closure, root hydraulic conductivity, and ROS detoxification (Riemann et al., 2015; Chhaya et al., 2021). Similarly, SA can enhance antioxidant activity and increase sensitivity to ABA, although high concentrations of SA can intensify oxidative stress or reduce beneficial effects, as observed in chickpeas under water stress (Kumar Patel et al., 2011; Salvi et al., 2021; Ahmad et al., 2023). These examples illustrate that the positive or negative outcome of hormonal signaling can vary depending on the hormone concentration, the timing of the response, the tissue and organ involved, and the intensity of the stress (Shaffique et al., 2023).

Other interactions are primarily antagonistic or related to growth and defense. Cytokinins typically act antagonistically to ABA in guard cells by reducing ABA sensitivity and promoting stomatal opening. This can help maintain photosynthesis under moderate stress but could compromise water conservation under severe drought (Hai et al., 2020). Similarly, drought-induced reductions in GA levels favor the stabilization of DELLA, thereby limiting vegetative growth and prioritizing protection against stress (Sarwar et al., 2023; Liao et al., 2025). Responses to auxins are also highly context-dependent, as auxins contribute to root growth and water search, but their spatial distribution and interaction with ABA and ethylene can modify root architecture under water-deficit conditions (Dinneny, 2019; Chhaya et al., 2021).

In legumes, drought signaling is intrinsically linked to symbiotic N_2_ fixation, as water deficit affects not only the roots and shoots but also nodule function. Moreover, drought responses must be coordinated with symbiotic N_2_ fixation and nodule maintenance (Etesami and Santoyo, 2025). Hormones such as ABA, ethylene, and cytokinins play critical roles in regulating nodule activity, senescence, and stress responses. This highlights the importance of integrating drought signaling with nodule functionality and symbiotic performance (Berrabah et al., 2024; da Silva et al., 2024; Ferguson et al., 2019). In a recent review, Etesami and Santoyo (2025) highlighted that ABA and ethylene are among the hormonal signals that can negatively affect nodule formation and development, while nodule non-rhizobial bacteria may partly counteract these limitations by reducing ethylene-mediated inhibition of nodulation.

Despite advances in drought stress signaling, legume-specific evidence remains fragmented, and future studies should clarify how hormonal communication interacts with symbiotic nitrogen fixation and nodule longevity, and how this differs among species, developmental stages, and drought intensities.

## 4 Responses to drought in legumes

Accessions of various legumes have developed sophisticated mechanisms to cope with drought, enabling them to survive water scarcity across both short- and long-term durations. These mechanisms can be summarized into three primary strategies: escape, avoidance, and tolerance (Hussain et al., 2019).

When facing water deficits, some plants respond by shortening their life cycle, a strategy known as the escape mechanism. Through rapid development, early flowering, and self-reproduction, these plants seek to complete their life cycle before the peak of the dry season. However, this early maturity usually comes at the expense of limited vegetative growth and a subsequent reduction in yield traits (Seleiman et al., 2021). The escape mechanism is particularly advantageous in environments where drought at the reproductive stages is recurrent. Thus, early-maturing genotypes can mitigate yield losses by shortening their developmental stages, effectively evading the drought period (Farooq et al., 2017). For instance, this strategy has been well-documented in cowpeas and groundnuts (Clavel et al., 2005; Hall, 2012). In cool-season grain legumes, such as lentils, drought escape is likewise associated with earlier flowering and maturity, especially under terminal drought scenarios, though this response involves trade-offs with biomass accumulation and seed filling (Noor et al., 2024).

Drought avoidance involves maintaining high plant water potential by enacting morphological and physiological changes while drought conditions persist. There are two primary avoidance strategies: the water-spender and the water-saver (Seleiman et al., 2021). *Water-spenders* circumvent cellular dehydration by maintaining high water uptake. This strategy relies on developing profuse, deep root systems to reach lower soil moisture reserves, albeit at a higher metabolic cost. Prolific and dense root systems are more effective for water uptake when surface soil moisture is depleted. Recent studies also emphasize the role of root hydraulic properties, aquaporin-regulated water transport, and increased stomatal sensitivity in determining the effectiveness of drought avoidance (Cardoso et al., 2025). Conversely, *water-savers* rely on sustaining water potential by strictly limiting the amount of water lost through transpiration. Beyond stomatal closure, xeromorphic adaptations, such as specialized leaf structures, are among the most efficient phenotypic traits for sustaining high water potential during scarcity (Seleiman et al., 2021). Cuticular wax, the primary component of the plant cuticle, acts as a hydrophobic barrier that regulates gas exchange and prevents non-stomatal water loss (Xue et al., 2017). In several species, drought adaptation includes the development of thick cuticles through increased wax deposition (Ilyas et al., 2021; Tomasi et al., 2024). In legumes, water-saver and water-spender genotypes have been found in common beans (Polania et al., 2016), faba beans (Belachew et al., 2019), cowpeas (Nunes et al., 2022), and lentils (Fernandez-Gutierrez et al., 2025).

While avoidance strategies may facilitate plant survival, seed production, and the completion of the life cycle in the mid-term, these adaptations often prove insufficient under prolonged drought (Osmolovskaya et al., 2018). When water deficit persists, more robust tolerance mechanisms must be activated to ensure survival (Seleiman et al., 2021). These mechanisms involve the induction of complex molecular pathways and the tight regulation of various phytohormones and signaling molecules. Central to this response is ABA, which acts as a regulatory hub coordinating stomatal behavior and transcriptional reprogramming via PYR/PYL/RCAR-PP2C-SnRK2 signaling. Both ABA-dependent and ABA-independent pathways ultimately converge on key transcription factors, including AREB/ABF, DREB, NAC, MYB, and WRKY (Cutler et al., 2010; Nakashima and Yamaguchi-Shinozaki, 2013; Yoshida et al., 2014; Zha et al., 2025).

The tolerance strategy also encompasses robust antioxidant mechanisms and osmotic adjustments. One of the most damaging consequences of cellular water stress is the overproduction of ROS. Their accumulation triggers oxidative damage across all cellular structures and macromolecules, which can eventually lead to programmed cell death. To achieve long-term tolerance, plants must develop efficient antioxidant defense systems, which consist of both enzymatic and non-enzymatic components. Enzymatic antioxidants include catalase, superoxide dismutase, glutathione peroxidase, and ascorbate peroxidase, alongside reductases such as glutathione reductase, dehydroascorbate reductase, and monodehydroascorbate reductase (Nadeem et al., 2019). These enzymes catalyze the stepwise transformation of ROS into less reactive intermediates and, ultimately, into water. In particular, the ascorbate-glutathione cycle is a primary redox-buffering system essential for ROS detoxification and drought tolerance (Foyer and Kunert, 2024). Enhanced activity of these antioxidant enzymes has been identified in drought-tolerant accessions of several legumes, including peas (Osman, 2015), chickpeas (Kumar Patel et al., 2011), soybeans (Guler and Pehlivan, 2016), and lentils (Sinha et al., 2018). Among the non-enzymatic components are a diverse array of molecules, such as ascorbic acid, polyamines, β-carotene, or α-tocopherol. Additionally, phenolic compounds and flavonoids contribute substantially to ROS scavenging and membrane protection under stress (Noor et al., 2024).

Another primary mechanism involved in the drought tolerance strategy is osmotic adjustment. Under drought conditions, certain plants activate the synthesis and accumulation of osmolytes - organic and inorganic solutes - within the cytoplasm. This process maintains a lower osmotic potential inside the cell relative to the outside environment, thereby sustaining the gradient necessary for water influx (Ilić et al., 2025). These compatible solutes, primarily carbohydrates and amino acids, also serve as protective molecules that preserve enzymatic activity and membrane stability (Khatun et al., 2021). Recent evidence further suggests that low-molecular-weight carbohydrates, including raffinose family oligosaccharides and sugar alcohols, may also contribute to drought adaptation in lentils and other related species (Dempsey and Thavarajah, 2024).

Beyond maintaining turgor, some of these solutes play a role in ROS detoxification. Among these compatible solutes, proline plays a central role in drought stress tolerance, acting as a multifunctional metabolite. It serves as an osmoprotectant and also functions as a signaling molecule in cell proliferation and mitochondrial regulation by triggering the activation of specific stress-responsive genes (Hussain et al., 2019; Jurkonienė et al., 2025). Consequently, proline is increasingly recognized for its role in redox buffering, stress recovery, and systemic signaling under prolonged water deficit (Haghpanah et al., 2024). Solute accumulation has been well documented in several legume crops, such as cowpeas, lentils, and chickpeas, as a physiological response to drought (Barbosa et al., 2013; Sinha et al., 2018; Priya et al., 2025). The main osmolytes that accumulate under drought conditions in chickpeas and common beans typically include inositol and sorbitol (Nadeem et al., 2019).

## 5 Evaluating drought tolerance

**Figure 3:**
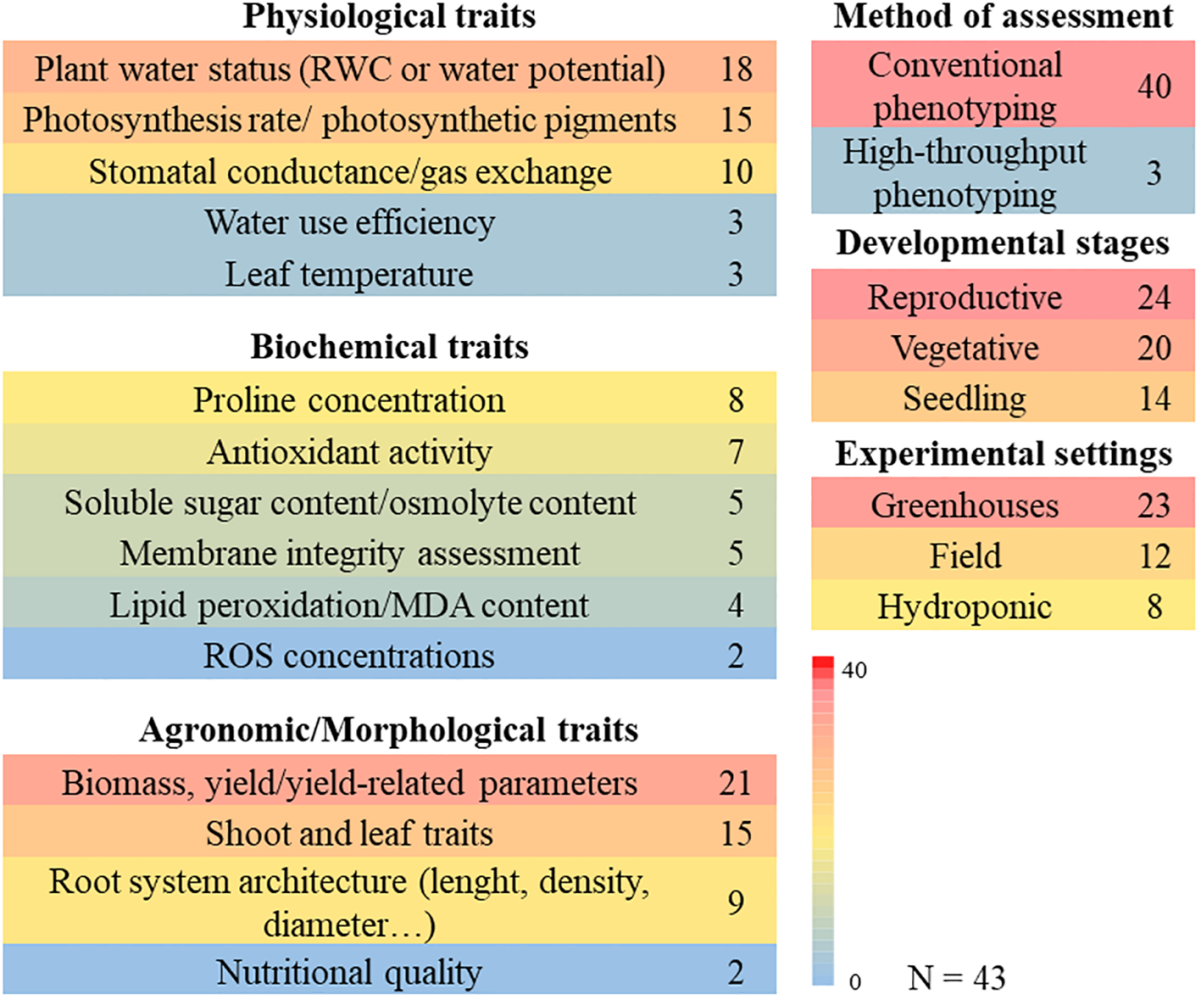
Graphical illustration of the different experimental parameters, configurations, and developmental stages used to evaluate drought tolerance in legumes. The tables summarize the information compiled from 43 studies, selected for their novelty, quality, and representativeness among peer-reviewed publications. The studies were selected from the Google Scholar database using the keywords: ‘drought’, ‘assessment’ and ‘legume’. Only original research articles were considered, selecting those records that sought to identify the intensity of stress in one or more legume species caused by water deficit. The experimental configurations of the selected records are summarized and represented in this figure. The numbers indicate the quantity of studies within each parameter or condition, and the colors represent the frequency of occurrence of each topic on a scale from 0 (light blue) to 40 (red). Authors’ own elaboration.

Breeding for drought-tolerant accessions begins with the rigorous assessment and identification of tolerant genotypes. An ideal phenotyping technique must be reliable, cost-effective, and ideally high-throughput; furthermore, the measured parameters should respond proportionally to stress levels and align with the experimental design (Moshelion et al., 2024). Drought experiments must also adhere to reproducible and standardized protocols. However, the variability observed across different drought evaluations in legumes highlights the urgent need for better standardization. This includes addressing discrepancies in tissue selection across diverse developmental stages, the specific parameters used to quantify tolerance levels, and the overall experimental settings. **Figure 3** summarizes a composite graph of the main experimental parameters, configurations, and developmental stages used to evaluate drought tolerance in legumes.

### 5.1 Developmental stages

The array of physiological and morphological responses in plants varies significantly depending on the developmental stage at which drought stress is imposed. Seedling, flowering, and pod-filling stages have all been utilized to study tolerance to water deficits in legume crops (Akter et al., 2021; Sinha et al., 2018).

On the one hand, screening and selecting tolerant accessions at early developmental stages, such as the seedling stage, can accelerate breeding programs and reduce operational costs. The experimental conditions required for assessing seedling drought tolerance allow for the evaluation of large populations in confined spaces at a relatively low cost, which is highly advantageous for identifying promising candidates within extensive germplasm collections (Ru et al., 2015). However, the outcomes of these controlled, early-stage experiments do not always correlate with yield traits measured under fluctuating field conditions (Akter et al., 2021; Sachdeva et al., 2022).

Conversely, drought stress imposed during reproductive stages is more likely to directly affect flower and seed formation (Gutierrez-Gonzalez et al., 2010; Jha et al., 2020), thereby providing a more accurate reflection of potential yield losses. Evaluating genotypes across the entire life cycle requires substantial time, labor, and financial resources, which often restricts the number of genotypes that can be analyzed simultaneously. Nevertheless, because the severity of water stress during the reproduction stages they are preferred for drought evaluations in legumes (**Figure 3**).

In legumes, the relationship between water stress responses at the seedling and reproductive stages remains poorly understood (Akter et al., 2021). Indeed, certain chickpea genotypes have been shown to respond differently to water deficits depending on the timing of stress imposition (Sachdeva et al., 2022). Consequently, screening experiments for tolerance assessment must be meticulously designed, and the developmental stage targeted for drought imposition must be selected based on the specific objectives of the breeding program.

### 5.2 Experimental settings

Traditionally, three main settings have been used for drought evaluations in legumes: hydroponic systems, greenhouses, and field conditions. In all these methods, the central challenge remains balancing control over natural environmental variables with the need to accurately simulate real-world drought periods. Experiments conducted in greenhouses are the predominant for assessing drought in legumes (**Figure 3**).

In hydroponic systems, plants are grown in nutrient-rich solutions without soil, which allows researchers to exercise precise control over water availability. Drought stress is typically simulated by adding osmotic agents such as polyethylene glycol (PEG) 6000 to the solution, a method successfully employed in studies of lentils and chickpeas (Muscolo et al., 2014; Yücel et al., 2010), or by directly exposing roots to air to reduce water availability (Singh et al., 2016).

In greenhouses, researchers simulate drought by strictly regulating irrigation. This setting has been fundamental for studying the morphological and physiological changes of legumes such as soybeans, lentils, and cowpeas (Barbosa et al., 2013; Fernandez-Gutierrez et al., 2025, 2026a);. Greenhouse experiments allow for the large-scale evaluation of numerous genotypes and facilitate repeated measurements under consistent conditions over time. However, the artificial nature of the environment and the limited size of the pots can restrict normal root growth or fail to fully mimic the complex dynamics of the field.

Field experiments provide the most realistic assessment of plant performance under natural conditions by incorporating random variations in temperature, light, and soil quality. They allow for the evaluation of genotype adaptation in real-world scenarios, including interactions between the plant and its natural ecosystem. Despite their realism, field trials have several drawbacks: the inability to perfectly replicate stress levels across different years and locations, the difficulty of disentangling genetic effects from environmental noise, and the need for space and resources, which limits the number of genotypes that can be evaluated. This approach has been widely used to assess various legumes, including common beans, cowpeas, and lentils (Akter et al., 2021; Burridge et al., 2016).

### 5.3 Parameters utilized for evaluating plant drought tolerance

#### 5.3.1 Conventional parameters

Evaluating drought tolerance requires measuring specific parameters to determine a plant’s overall fitness and its ability to respond to stress. Various physiological, biochemical, and agronomic parameters are used to evaluate drought tolerance in legumes, reflecting the plant’s capacity to initially withstand and subsequently recover from water deficits.

Agronomic traits, such as biomass accumulation and yield components, are frequently used as indicators of water stress severity. Total aboveground biomass, as well as the biomass of specific organs (e.g., leaves or stems), provides critical insights into the plant’s ability to maintain growth and productivity under water deficit (Yücel et al., 2010). Accessions that maintain high yields under drought conditions are generally classified as tolerant. Key metrics for gauging the impact of drought on production include the number of seeds, seed size (often estimated by the weight of 100 seeds), pods per plant, and total seed weight (Akter et al., 2021). These yield estimates have been fundamental for selecting tolerant accessions in major legume crops such as lentils and soybeans (Fernandez-Gutierrez et al., 2026a, b; Yan et al., 2020).

Furthermore, quantitative and qualitative assessments of root system architecture including root length, density, and spatial distribution are essential for evaluating drought tolerance (Akter et al., 2021; Priya et al., 2021). Root-related parameters provide direct insight on water uptake efficiency. For instance, in soybeans, traits such as root surface area have demonstrated high heritability and contribute significantly to drought tolerance, especially during the vegetative stage (Yan et al., 2020). Similarly, root length density has been positively correlated with higher yields under drought conditions in chickpeas, although its heritability can vary significantly (Varshney et al., 2014).

Plant water status provides crucial information on how plants physiologically adapt to water deficits (Feng et al., 2022). For example, relative water content (RWC) reflects the water-holding capacity of plant tissues and serves as a reliable indicator of internal dehydration levels. In legume crops, higher RWC under stress has been linked to superior osmoregulation and enhanced membrane stability (Dash et al., 2020; Fernandez-Gutierrez et al., 2026a). Similarly, leaf and xylem water potential are reliable indicators of hydrological status. As soil moisture declines, plant water potential drops, which can lead to xylem embolism and desiccation. Therefore, the ability of certain accessions to maintain higher water potentials under drought is a key determinant for survival (Nunes et al., 2022).

Stomatal closure is the primary response to water deficit, and thus, regulating its opening and closing is essential for balancing photosynthesis with survival. The degree and duration of stomatal closure can effectively differentiate genotypes according to their drought tolerance strategies. Stomatal conductance reflects the degree of closure and serves as an indicator of the plant’s water-saving capacity (Buckley et al., 2025). This parameter is widely used to assess tolerance in legumes such as common beans and alfalfa (Polania et al., 2022; Prince et al., 2022). Furthermore, since decreased conductance is directly correlated with increased leaf temperature, thermography has become a valuable tool for the non-destructive assessment of stress (Djanaguiraman et al., 2023; Frey et al., 2023).

Both photosynthetic rate (A) and water use efficiency (WUE) are significantly altered under drought conditions (Tovignan et al., 2023). While A indicates the capacity to assimilate C under water-limited conditions, WUE measures the efficiency of converting water into biomass, generally calculated by dividing the aboveground biomass yield, measured as yield weight, by the amount of water used. Drought-sensitive accessions typically exhibit a more pronounced decline in photosynthetic rates than tolerant ones. Furthermore, since chlorophyll degrades as drought progresses, measurements of leaf chlorophyll content and chlorophyll fluorescence (e.g., F_v_/F_m_) provide valuable information on the integrity of the photosynthetic apparatus (Dash et al., 2020; Yan et al., 2024).

Other key metrics include the quantification of oxidative stress and osmolyte accumulation. The intensity of oxidative damage can be estimated by measuring the activity of antioxidant enzymes, ROS concentrations (such as H_2_O_2_), or lipid peroxidation via malondialdehyde content (Sinha et al., 2018). Conversely, osmotic adjustment is quantified by soluble sugar content or proline concentration, which acts as both an osmolyte and antioxidant (Sinha et al., 2018). Finally, electrolyte leakage and the membrane injury index are commonly measured to evaluate cell membrane integrity, revealing a genotype’s fundamental tolerance to dehydration (Mishra et al., 2018).

#### 5.3.2 High-throughput drought tolerance assessment

When evaluating large number of plants, conventional methods can be replaced by high-throughput phenotyping (HTP). The development of second- and third-generation sequencers capable of generating massive amounts of genomic data cost-effectively, has highlighted the urgent need for similarly efficient phenotyping. To avoid research bottlenecks, phenotypic evaluations must be as rapid, accurate, and cost-effective as modern genotyping. HTP approaches are particularly effective for estimating complex biotic and abiotic stresses, including drought tolerance, where the multifaceted nature of traits makes conventional phenotyping challenging and time-consuming.

The tools used for HTP can be classified into two main groups. The first comprises infrared spectroscopy techniques, which quantify the nutritional traits of seeds with high precision. The most established systems include FT-NIR (Fourier Transform Near-Infrared) and FT-MIR (Fourier Transform Mid-Infrared) spectroscopies. While FT-NIR has been used to evaluate protein content in peas and lentils (Hang et al., 2022), more recent approaches favor FT-MIR due to its greater accuracy. FT-MIR has been successfully employed for dietary fiber quantification in pulse crops such as chickpeas, dry peas, and lentils (Madurapperumage et al., 2025). These technologies, although accurate for nutritional assessment, are not fully related to the phenotyping of drought tolerance itself.

The second group, comprised by the temporal imaging systems, includes unmanned aerial systems (UASs), the most common being unmanned aerial vehicles (UAVs) used to measure agronomic traits. UASs can be integrated with various sensors, such as RGB, multispectral, hyperspectral, thermal, radio detection and ranging (RADAR), and light detection and ranging (LiDAR) cameras, to measure traits like plant height, days to flowering, days to maturity, lodging, and disease incidence. These imaging systems encompass the most commonly used HTP traits for drought tolerance phenotyping. The simplest assessment of water stress is performed using RGB sensors that can detect changes in the structure, height and shape of the plant canopy. The combination of RBG and infrared imaging enabled the development of the normalized difference vegetation index (NDVI), which accurately predicts vegetation health (Condorelli et al., 2018). Hyperspectral sensors can measure the rate of photosynthesis, providing a precise estimate of plant fitness, and thermal images capture canopy temperature and transpiration rates (Kim et al., 2021).

It is worth noting that HTP devices can be sensitive to environmental conditions in the field, which can negatively affect data reproducibility (Machwitz et al., 2021). For example, wind can interfere with drone calibration and measurements, while thermography is often affected by canopy shading and background thermal interference (Angidi et al., 2025).

Similar to high-throughput genotyping, the monitoring of plants via HTP generates a massive volume of data that is difficult to capture or process using conventional methods. Screening thousands of plants in large-scale breeding plots allows for the detection of subtle variations in traits and facilitates the early detection of stress, providing highly informative datasets for researchers. Regardless of the specific HTP approach employed, the resulting data must be stored and processed using advanced computational resources. To this end, Machine Learning (ML) and other Artificial Intelligence (AI) algorithms are being developed to analyze and discriminate between complex phenotypic datasets in an accessible and efficient manner. These AI systems are rapidly evolving to analyze the large amount of data obtained from HTP sensors and platforms. However, the integration of these data is still underdeveloped and generally lacks a biological translation (Angidi et al., 2025). The considerable financial investment required to implement an HTP station often hinders its adoption by smaller research groups or farmers. Furthermore, the lack of standardization among diverse HTP platforms remains a significant challenge, complicating the comparison of results between different crops or experimental settings (Xu et al., 2015).

In legumes, HTP is expected to transform traditional phenotyping through the implementation of non-destructive monitoring techniques. These advancements are very promising for agricultural and nutritional monitoring, as they facilitate the rapid identification of optimal parental donors for breeding programs. Specifically for water stress assessment, HTP has been proposed as a robust tool for precise selection in major legume crops, including chickpeas, common beans, and pigeon peas (Javornik et al., 2025; Pappula-Reddy et al., 2024; Prasad et al., 2026). However, few experiments have successfully applied HTP to the identification of QTLs/markers in legumes (**Figure 3**). For example, drought-tolerant genotypes have been detected using high-throughput imaging platforms in Phaseolinae (Verheyen et al., 2024), tepary beans (Leal-Delgado et al., 2019), and peas (Bagheri et al., 2026); and through UAV-based monitoring in dry beans (Sankaran et al., 2018) and peanuts (Bagherian et al., 2023).

## 6 Discussion

Drought is recognized as one of the most devastating abiotic stresses impacting legume production worldwide (Pérez de la Vega et al., 2022; Khatun et al., 2021). In the context of climate change, the genetic improvement of drought-tolerant varieties has become a fundamental priority for sustaining agricultural yields and ensuring global food security. The ability to accurately discriminate between genotypes based on their tolerance to water deficit facilitates the deployment of resilient crops and supports the development of breeding programs aimed at introgressing drought-tolerant traits into widely cultivated but sensitive varieties (Akter et al., 2021; Varshney et al., 2021). Yet, drought tolerance is often misinterpreted, as different studies may consider entirely different tolerance mechanisms. For example, genotypes with an escape strategy early in their development are often classified as drought-tolerant, even though their strategy is based on stress avoidance. Therefore, before starting a breeding program, it is essential to define drought tolerance itself. This review analyzes drought tolerance as a mechanism involving long-term survival and the activation of stress-related genes. Further empirical studies are urgently needed to elucidate the regulatory pathways governing these responses.

An important feature when studying drought tolerance is the multifaceted nature of this abiotic stress. While numerous physiological, biochemical, and molecular pathways associated with drought adaptation have been identified (Varshney et al., 2021), these mechanisms are often investigated independently, resulting in a limited understanding of drought tolerance as a whole. Drought tolerance is not controlled by a single mechanism but rather by a dynamic integration of strategies and responses. Furthermore, some drought responses remain controversial and require further investigation, such as the specific role of stomata in drought tolerance, which is not yet fully understood. Although stomatal closure has been established as one of the first responses to water deficit, the precise mechanisms that regulate stomatal behavior over extended periods, balancing CO_2_ assimilation with water loss through transpiration, remain unclear (Abdalla et al., 2021; Buckley, 2019; Cardoso et al., 2025; Carminati and Javaux, 2020).

Even though substantial progress has been made in understanding plant responses to drought, the translation of this knowledge into the development of drought-resilient legume cultivars remains limited. This challenge is largely driven by the still poorly explored adaptation of the mechanisms underlying drought tolerance to legume species. Furthermore, most of the current knowledge derives from soybean studies, lacking verification in other legume crops. There are significant gaps in characterizing the role of symbiotic interactions in drought tolerance and in identifying the regulatory networks that control these interactions under water deficit conditions.

In legumes, drought responses cannot be fully understood independently of rhizobia and AMF (Etesami and Santoyo, 2025; Hu et al., 2022). These symbiosis influences not only nutrient acquisition and water uptake but also hormonal signaling, antioxidant metabolism, and gene expression in response to stress. Growing evidence supporting synergistic interactions between rhizobia and AMF suggests that drought tolerance may arise not only from plant genetics but also from the influence of the entire microbiome surrounding the root system. This perspective challenges traditional breeding approaches focused exclusively on plant traits and highlights the need to incorporate microbial interactions into future drought resilience strategies for legumes.

Although ABA signaling pathways have been considered central to drought responses, increasing evidence suggests that drought adaptation cannot be explained solely by ABA accumulation and stomatal regulation. Hormonal interactions involving jasmonates, salicylic acid, ethylene, auxins, cytokinins and gibberellins generate complex regulatory networks. Observations such as the differences in the effect of varying doses of SA (which enhances antioxidant defenses at low concentrations while inducing oxidative stress at higher doses) reveal the delicate balance of phytohormones necessary to adequately articulate drought tolerance. This complex network of phytohormones is even more intricate in legumes, where they play a crucial role in nodule regulation, which in turn contribute to the plant’s fitness under stress (Berrabah et al., 2024). Despite all this knowledge, little of this evidence has been studied in legumes, which represents a crucial gap in clarifying the drought tolerance strategy in these specific crops.

In recent years, several legume-specific advances have broadened the understanding of drought tolerance/resistance beyond the canonical ABA-mediated pathways. Current advances emphasize root architectural traits that enhance water acquisition, nodulation maintenance, and biological N_2_ fixation under water deficit, and the use of HTP to detect adaptative variation to drought among genotypes (Priya et al., 2021; Iqbal et al., 2022; da Silva et al., 2024; Pappula-Reddy et al., 2024; Afonso et al., 2025; Rubia et al., 2025). Along with recent reviews on lentil adaptation and panomics-assisted legume improvement, these studies indicate that drought research in legumes is increasingly integrating root traits, nodulation and N_2_ fixation, phenomics, and multi-omics approaches to identify resistance mechanisms relevant to genetic improvement (Noor et al., 2024; Hu et al., 2025).

Another major limitation in current drought research is the predominant reliance on controlled experimental systems (Osmolovskaya et al., 2018). Hydroponic and greenhouse studies offer high experimental reproducibility and facilitate mechanistic analyses; however, these systems often fail to replicate the complexity of field drought scenarios, where plants experience simultaneous fluctuations in temperature, radiation, soil heterogeneity, nutrient availability, and biotic interactions. Consequently, drought response traits identified under artificial conditions may have low predictive value in agricultural settings. This discrepancy is particularly evident when comparing the evaluation at the seedling stage with the performance at the reproductive stage, since tolerance during early stages of development usually shows a weak correlation with yield traits at maturity.

The lack of standardized phenotyping methodologies further complicates comparisons between studies (Moshelion et al., 2024). Variability in stress imposition protocols, developmental stage, tissue selection, and assessed parameters leads to inconsistent conclusions. Furthermore, drought tolerance is frequently inferred from isolated physiological traits without considering their integration into overall plant performance. For instance, high water use efficiency or reduced stomatal conductance do not necessarily translate into improved yield under prolonged drought. This methodological fragmentation continues to hinder the identification of universally reliable drought tolerance markers for legumes. To address this problem, analyzing correlations between different phenotypic parameters and establishing standardized methodologies would facilitate more reliable comparisons across independent studies (Moshelion et al., 2024).

The emergence of analytical tools based on HTP and AI has been proposed as a transformative approach to improving phenotypic evaluation, significantly increasing the accuracy of measurements and enabling large-scale evaluation of diverse genotypes, while reducing the time and labor required for data collection (Javornik et al., 2025; Pappula-Reddy et al., 2024; Prasad et al., 2026). These technologies enhance phenotyping capabilities and allow for the integration of complex temporal and spatial datasets. However, several significant challenges remain, including high sensitivity to ambient noise (which may compromise reproducibility), data integration and translation into biological understanding, and the high infrastructural and computational costs associated with HTP systems. Therefore, while the integration of HTP and AI provides a powerful framework for accelerating plant breeding programs in increasingly challenging future climatic scenarios, its success will depend on the development of standardized and biologically interpretable methods, as well as on reducing implementation costs.

In general, future progress in drought research in legumes requires an integrated approach that considers the full complexity of drought tolerance traits. Combining research in physiology, genomics, phenomics, microbiome, and envirotyping under realistic environmental conditions can provide a more accurate understanding of drought adaptation. Rather than identifying isolated “tolerance traits”, future breeding programs should prioritize dynamic trait networks capable of maintaining productivity under heterogeneous and unpredictable drought scenarios. The success of these integrated approaches will depend on the implementation and expansion of HTP technologies that overcome human limitations in the context of plant phenotyping. Combining novel HTP technologies with a comprehensive understanding of responses to water stress will facilitate the development of next-generation legume cultivars with greater resilience to water-scarce environments, ensuring stable production under future climate scenarios.

## 7 Conclusion and future perspectives

Drought tolerance in legumes is a complex trait resulting from the interaction of physiological, molecular, hormonal, and symbiotic mechanisms, rather than isolated responses. While significant progress has been made in understanding plant responses to drought, substantial gaps remain in our overall knowledge, particularly in legumes. Furthermore, the lack of standardized phenotyping methodologies and the limited transferability of controlled-condition studies to field environments continue to constrain the progress of plant breeding.

Future research should adopt integrative approaches that combine physiology, genomics, phenomics, microbiome studies, and field analyses to better understand drought adaptation under real-world agricultural conditions. Special attention should be paid to nodulation and symbiotic nitrogen fixation. These factors appear to play a fundamental role in the resistance of legumes to water deficit. The ability to maintain functional nodules, efficient rhizobial associations, and coordinated responses between the plant, microbiome, and hormones in the event of drought may be key to ensuring legume resilience and yield stability under water-scarce conditions.

Finally, the future implementation of HTP and AI-based tools, as well as their integration into breeding programs, could substantially accelerate the identification of drought-resistant genotypes. However, it is important to emphasize that these technologies are still under development, and their success will depend on methodological standardization, biological interpretability, and greater accessibility. Together, these advances will contribute to the development of next-generation legume cultivars better adapted to future climate scenarios.
